# Trastuzumab-DM1 causes tumour growth inhibition by mitotic catastrophe in trastuzumab-resistant breast cancer cells *in vivo*

**DOI:** 10.1186/bcr2868

**Published:** 2011-04-21

**Authors:** Mark Barok, Minna Tanner, Katri Köninki, Jorma Isola

**Affiliations:** 1 Institute of Medical Technology, University of Tampere, Tampere, Biokatu 6, Tampere 33014, Finland; 2 Department of Oncology, University Hospital of Tampere, Teiskontie 35, Tampere 33520, Finland

## Abstract

**Introduction:**

Trastuzumab is widely used for the treatment of HER2-positive breast cancer. Despite encouraging clinical results, a significant fraction of patients are, or become, refractory to the drug. To overcome this, trastuzumab-DM1 (T-DM1), a newer, more potent drug has been introduced. We tested the efficacy and mechanisms of action of T-DM1 in nine HER2-positive breast cancer cell lines *in vitro *and *in vivo*. The nine cell lines studied included UACC-893, MDA-453 and JIMT-1, which are resistant to both trastuzumab and lapatinib.

**Methods:**

AlamarBlue cell-proliferation assay was used to determine the growth response of breast cancer cell lines to trastuzumab and T-DM1 *in vitro*. Trastuzumab- and T-DM1-mediated antibody-dependent cellular cytotoxicity (ADCC) was analysed by measuring the lactate dehydrogenase released from the cancer cells as a result of ADCC activity of peripheral blood mononuclear cells. Severe Combined Immunodeficient (SCID) mice were inoculated with trastuzumab-resistant JIMT-1 cells to investigate the tumour inhibitory effect of T-DM1 *in vivo*. The xenograft samples were investigated using histology and immunohistochemistry.

**Results:**

T-DM1 was strongly growth inhibitory on all investigated HER2-positive breast cancer cell lines *in vitro*. T-DM1 also evoked antibody-dependent cellular cytotoxicity (ADCC) similar to that of trastuzumab. Outgrowth of JIMT-1 xenograft tumours in SCID mice was significantly inhibited by T-DM1. Histologically, the cellular response to T-DM1 consisted of apoptosis and mitotic catastrophe, the latter evidenced by an increased number of cells with aberrant mitotic figures and giant multinucleated cells.

**Conclusions:**

Our results suggest mitotic catastrophe as a previously undescribed mechanism of action of T-DM1. T-DM1 was found effective even on breast cancer cell lines with moderate HER2 expression levels and cross-resistance to trastuzumab and lapatinib (MDA-453 and JIMT-1).

## Introduction

HER2 (ErbB2) is a member of the epidermal growth factor receptor (EGFR) family of receptor tyrosine kinases. Its overexpression occurs in 15 to 20% of primary human breast cancers and is associated with aggressive growth and poor clinical outcomes [1,2]. A breakthrough in medical oncology was the finding that trastuzumab, a recombinant humanized monoclonal antibody against the extracellular domain of HER2, showed a significant anti-tumour effect in a phase III clinical trial [3]. Trastuzumab is currently used for treatment of both metastatic and early-stage breast cancer world-wide [3,4].

Although the mechanisms underlying the action of trastuzumab are still not fully determined, its clinical benefit is attributed to internalization and down-regulation of cell surface HER2 [5], preventing the activation of AKT by reducing signaling in the PI3K-PTEN pathway [6], cell cycle arrest in G_1 _[7], HLA-I-restricted antigen presentation of HER2 [8], inhibition of angiogenesis [9] and evoking antibody-dependent cellular cytotoxicity (ADCC) [10,11]. In spite of these multiple actions, a significant number of breast cancer patients are primarily resistant to trastuzumab, and a majority of those initially responding become resistant during prolonged treatment [12]. Primary or secondary resistance to trastuzumab is attributed to autocrine production of EGF-related ligands [13], activation of the insulin-like growth factor-I (IGF-I) receptor pathway [14], defects in the PI3K-PTEN-AKT pathway [6,15], masking of the trastuzumab epitope by MUC4 [16] or hyaluronan [17], expression of p95HER2, a constitutively active truncated form of HER2 [18], or impaired ADCC reaction [19].

Since development of trastuzumab, several other drugs targeting the HER receptor family, have been developed [20,21]. Of those, a small molecule kinase inhibitor lapatinib has proven effective in clinical trials [22,23]. Unfortunately, similar to trastuzumab, a majority of patients responding to lapatinib become resistant and most of trastuzumab-pretreated patients fail to respond to lapatinib [24]. Therefore, it is clear that more effective HER2 targeting drugs are needed.

A new strategy of anti-HER2 targeted therapy has been achieved using antibody-drug conjugate (ADC) technology. The monoclonal antibody trastuzumab has been conjugated with cytotoxic molecule DM1 (derivative of maytansine 1). The resulting conjugate, named trastuzumab-DM1 (T-DM1) is designed to deliver DM1 into the HER2 overexpressing cells via receptor-mediated endocytosis [25]. Active DM1 is released following internalization of the conjugate and lysosomal degradation [26]. Intracellular DM1 is a potent inhibitor of microtubule assembly thereby causing cell death [27-29]. T-DM1 is effective both *in vitro *and *in vivo *models of trastuzumab-resistant breast cancer [25]. Very recently, T-DM1 showed remarkable activity in phase I and II studies in which it was given to patients with trastuzumab-resistant HER2-positive breast cancer [30-32].

T-DM1 has been shown to induce apoptotic cell death [25]. Other microtubule inhibitors (such as taxanes) might also lead to mitotic catastrophe (MC), which can be observed histologically [33,34]. Using a trastuzumab resistant xenograft tumour model, we showed that T-DM1 can induce both apoptosis and mitotic catastrophe. The latter mechanism is described for T-DM1 for the first time.

## Materials and methods

### Cells

The human breast cancer cell line EFM-192A was obtained from the German Resource Center for Biological Material and the cell lines BT-474, MDA-361, MDA-453, MCF-7, SK-BR-3, UACC-812, UACC-893 and ZR-75-30 were obtained from the American Type Tissue Culture Collection. The JIMT-1 cell line has been established in the laboratory of Cancer Biology, University of Tampere, Finland [35] (also available via German Resource Center for Biological Material). The cell lines were cultured according to recommended specifications.

### Antibodies

Trastuzumab (Herceptin^®^) and rituximab (Mabthera^®^) were purchased from Roche Ltd. (Basel, Switzerland). Trastuzumab-DM1(T-DM1) was provided by Genentech Inc. (South San Francisco, CA, USA) through a Materials Transfer Agreement. Mouse M30 CytoDeath antibody was obtained from Roche Ltd., HercepTest staining kit was purchased from DakoCytomation (Carpinteria, CA, USA), rabbit monoclonal antibody against human HER2 (clone SP3) was obtained from NeoMarkers/Lab Vision (Fremont, CA, USA).

### *In vitro *assay of drug sensitivity

The effects of trastuzumab and T-DM1 on cell growth was examined by the AlamarBlue method (Invitrogen, Carlsbad, CA, USA). The cells were trypsinised and plated in 96-well, flat-bottomed, tissue culture plates. The effects of trastuzumab and T-DM1 were tested at a concentration of 0.001, 0.01, 0.1, 1, and 10 μg/ml. An MCF-7 HER2 negative breast cancer cell line with low trastuzumab binding capacity was used as a negative control. The number of viable cells was tested at 72 hours after drug exposure by adding the AlamarBlue reagent. Fluorescence was measured with excitation at 544 nm and emission at 590 nm using a Wallac Victor2 plate reader (Perkin-Elmer, Turku, Finland). Fluorescence values of samples were normalised with values of the cell culture media without cells. The results presented as the proportion of viable cells were calculated by dividing the fluorescence values of drug treated samples by the fluorescence values of untreated control samples.

### Measurement of antibody-dependent cellular cytotoxicity (ADCC)

ADCC was analysed by measuring the lactate dehydrogenase (LDH) released from the cancer cells as a result of ADCC activity of peripheral blood mononuclear cells (PBMC). PBMCs were separated from the heparinized blood of a single healthy donor by Ficoll density gradient centrifugation (Histopaque-1077, Sigma-Aldrich, St. Louis, MO. USA). Cancer cells (target; 5,000 to 10,000 per well) and PBMCs (effector) were co-incubated at 1:5, 1:10, 1:20, 1:40 and 1:80 target:effector ratios in 100 μL DMEM containing 5% FCS in a 96-well U-bottomed plate in quadruplicate for six hours at 37°C with trastuzumab, T-DM1 or negative control antibody, rituximab (20 μg/ml). ADCC was measured in a six-hour LDH release assay (CytoTox Non-Radioactive Cytotoxicity Assay, Promega Corporation, Madison, WI, USA). The absorbance at 490 nm was recorded by using a microplate reader (Model 680XR, Bio-Rad, Hercules, CA, USA). The negative control sample (target spontaneous) was prepared identically, contained trastuzumab or T-DM1 or rituximab, but did not contain PBMCs; effector spontaneous sample contained no target cells. Tumour cells killed by freezing at -80°C for one hour then warming up to 37°C served as positive control (target maximum). The percentage of cells killed was calculated according to the following formula: (experimental - effector spontaneous - target spontaneous)/(target maximum - target spontaneous) * 100.

The blood donor had given informed consent before for obtaining a peripheral venous blood sample for PBMC assays. These experiments were done according to the rules of the Ethical Committee of University Hospital of Tampere.

### *In vivo *assay of drug sensitivity

Five- to eight-week-old female SCID mice (Harlan Netherlands, Horst, Netherlands) were given a single subcutaneous injection of 5 × 10^6 ^JIMT-1 cells suspended in 100 μl cell culture medium (DMEM supplemented with 7.5% FBS). Rituximab (5 mg/kg) and trastuzumab (5 mg/kg) were given intraperitoneally (i.p.) once per week, weekly T-DM1 (5 or 15 mg/kg) was given intravenously (i.v.) as it has been shown to be an effective regimen by Lewis Phillips *et al. *[25]. Lapatinib powder was formulated prior to dosing every day in a vehicle of 0.5% hydroxypropyl methyl cellulose and 0.1% Tween 80 and administered orally as a suspension at a dose of 100 mg/kg for 34 days on a daily schedule [36]. Tumour growth was measured with a caliper and tumour volume was calculated using the formula *T*_vol _= *π/*6 × larger diameter × (smaller diameter)^2^. Animals were euthanized by CO_2 _inhalation. The experiments were done with the approval by the National Animal Experiment Board.

### Immunohistochemistry

Samples of xenograft tumours were fixed in 4% buffered formaldehyde for 24 h, processed into paraffin, then sectioned at 5 μm. Sections were deparaffinized and stained with hematoxylin and eosin (H&E). For immunohistochemistry, tissue sections were deparaffinised followed by antigen-retrieval in Tris-EDTA buffer (0.01 M pH 9.0) at high temperature (water bath, 30 minutes at 98°C). After blocking for non-specific binding, primary antibodies (see above) were applied at optimized concentrations and incubated (30 minutes at room temperature). Standard peroxidase-polymer kit (PowerVision+ poly-HRP IHC Detection Systems, Leica Biosystems Newcastle Ltd., Newcastle, UK) was used for visualisation, with diaminobenzidine as the chromogen (Vector Laboratories Inc., Burlingame, USA). Slide scanning was done using Aperio ScanScope XT at superresolution (40×).

Cells with normal and aberrant mitotic shape were defined morphologically as described earlier [37], giant multinucleated cells (GMC) were defined as cells with more than three nuclei. Cells with normal and aberrant mitotic shape and GMCs were counted in hematoxylin and eosin stained histological sections. Apoptotic cells were counted as positive cells using CytoDeath antibody immunohistochemistry [38]. Cells with normal and aberrant mitotic shape, GMCs and apoptotic cells were counted on a minimum of 10 randomly selected × 40 high-power fields containing representative sections of tumour. Data are presented as the average of positive cells ± SD/field.

### Statistical analysis

Data are expressed as the mean ± SE. The statistical significance of the differences between means were determined using Student's *t *test for two samples after verifying that data passed the normality test and the groups compared have equal variance. Unpaired groups were compared with the Mann-Whitney *U *test. Differences were statistically significant at *P *< 0.05.

## Results

### *In vitro *sensitivity of HER2 positive breast cancer cells to trastuzumab and T-DM1

We studied a panel of nine HER2 overexpressing breast cancer cell lines, which have been previously determined as sensitive to both trastuzumab and lapatinib (ZR-75-30, BT-474, EFM-192A, SKBR-3 UACC-812), sensitive to trastuzumab and resistant to lapatinib (MDA-361), or resistant to both trastuzumab and lapatinib (JIMT-1, UACC-893, MDA-453) [39].

**Figure 1:**
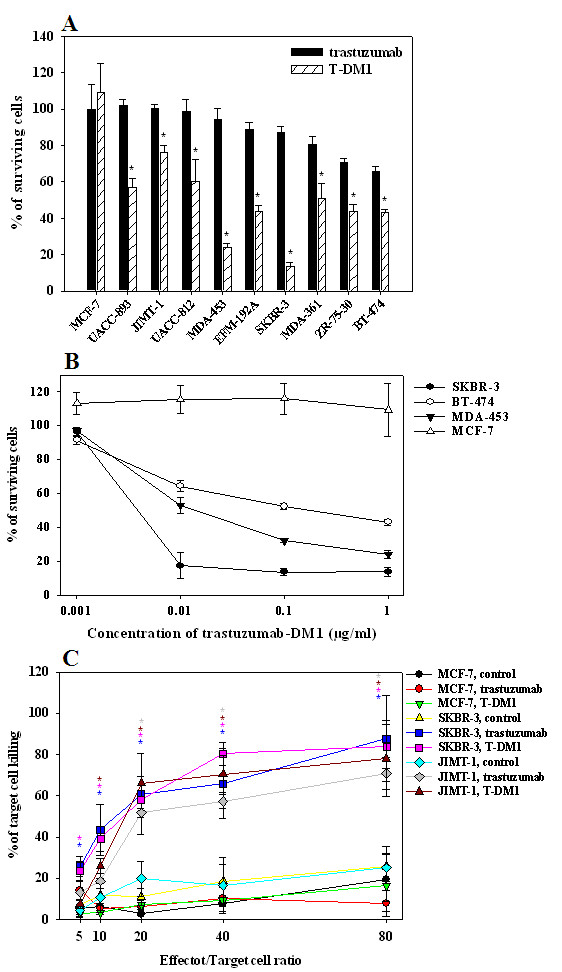
***In vitro *effects of trastuzumab and T-DM1**. **A**. Growth inhibitory effects of trastuzumab and T-DM1 on HER2 positive breast cancer cell lines *in vitro*. T-DM1 (1 μg/ml, hatched column) significantly inhibited the growth of all HER2 positive breast cancer cell lines in comparison to trastuzumab (10 μg/ml, black column) (*, *P *< 0.05). Neither trastuzumab nor T-DM1 had any growth inhibitory effect on MCF-7 cells with low trastuzumab binding capacity. Note that trastuzumab was used in a 10-times higher dose than T-DM1. **B**. T-DM1 inhibited the growth of HER2 positive breast cancer cells in a dose-dependent manner. The effect of T-DM1 was tested at a concentration of 0.001, 0.01, 0.1 and 1 μg/ml. Its dose-dependent growth inhibitory effect was seen on SKBR-3 (black circle), BT-474 (white circle) and MDA-453 (black triangle) HER2 positive breast cancer cell lines. No inhibition of HER2 negative MCF-7 (white triangle) breast cancer cells was seen. **C**. Trastuzumab and T-DM1 evoke similar efficient ADCC on HER2 positive breast cancer cells *in vitro*. Target tumour cells (SKBR-3, JIMT-1, MCF-7) were mixed with peripheral blood mononuclear cells (PBMCs) freshly isolated from peripheral blood at effector/target ratios of 5:1, 10:1, 20:1, 40:1, and 80:1. ADCC was analyzed by measuring the lactate dehydrogenase (LDH) released from the cancer cells as a result of ADCC activity of PBMCs in the presence of trastuzumab, T-DM1 or control antibody (rituximab) (20 μg/ml). The percentage of killed cells was calculated as described in Materials and methods. Killing of SKBR-3 cells was significantly higher in the presence of both trastuzumab (blue) and T-DM1 (pink) in comparison to that in the presence of control antibody (yellow) when the effector/target cell ratio was 5 or above (blue and pink stars, *P *< 0.05). Similarly, significantly higher killing of JIMT-1 cells was detected in the presence of both trastuzumab (gray) and T-DM1 (brown) compared to that in the presence of control antibody (pale blue) when the effector/target cell ratio was 20 or above (gray and brown stars, *P *< 0.05). Trastuzumab and T-DM1 evoked similar tumour cell killing on the HER2 positive breast cancer cell lines (SKBR-3 and JIMT-1). MCF-7 cells with weak trastuzumab binding capacity showed low levels of both trastuzumab- and T-DM1-evoked ADCC-mediated killing (red and green, respectively).

After 72 h incubation, T-DM1 significantly inhibited the growth of all HER2 positive cell lines compared to trastuzumab (*P *< 0.05). T-DM1 produced a more pronounced effect on trastuzumab- and lapatinib-sensitive cell lines (13 to 43% of surviving cells), but inhibitory effect was seen on MDA-361 cells (50 ± 8%) as well as on cell lines which are resistant to both trastuzumab and lapatinib (MDA-453, 23 ± 2%; UACC-893, 56 ± 4%; JIMT-1, 76 ± 4%). Interestingly, the trastuzumab- and lapatinib-resistant MDA-453 cell line was the second most sensitive to T-DM1. No inhibition was seen in HER2 negative control cell line (MCF-7) (Figure 1A.). T-DM1 inhibited the growth of HER2 positive breast cancer cells in a dose-dependent manner (Figure 1B.). Concentrations of T-DM1 higher than 1 μg/ml inhibited also the growth of the HER2 negative control cell line (MCF-7), suggesting a non-specific toxic effect (data not shown).

**Figure 2:**
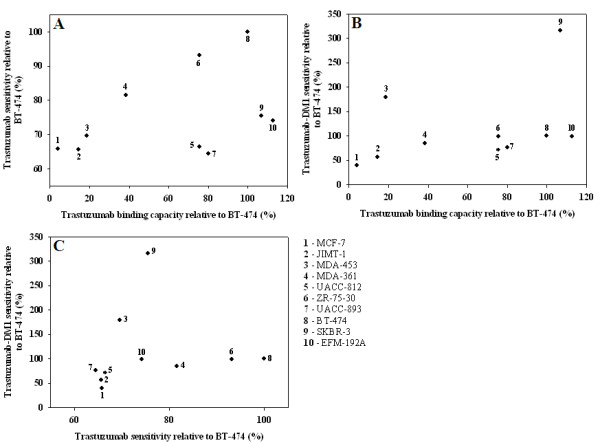
**Correlations of drug responses and trastuzumab binding capacities**. **A**. Correlation of response to trastuzumab and trastuzumab binding capacity. No significant correlation was found when trastuzumab binding capacities (determined by flow cytometry [40]) were plotted against trastuzumab sensitivities of the breast cancer cell lines (*R *= 0.43, *P *= 0.19). Trastuzumab sensitivities are expressed in proportion to the BT-474 cell line. **B**. Correlation of response to T-DM1 and trastuzumab binding capacity. No significant correlation was found when trastuzumab binding capacities were plotted against T-DM1 sensitivities of the breast cancer cell lines (*R *= 0.58, *P *= 0.06). T-DM1 sensitivities are expressed in proportion to the BT-474 cell line. **C**. Correlation of trastuzumab and T-DM1 responses. Effects of both drugs on breast cancer cell lines were plotted. No significant correlation was found between the responsiveness to trastuzumab and T-DM1 (*R *= 0.6, *P *= 0.07). Drug sensitivities are expressed in proportion to the BT-474 cell line.

Next we correlated the effects of trastuzumab and T-DM1 to the trastuzumab-binding capacity of each cell line [40]. No significant correlations were found (*R *= 0.43, *P *= 0.19 and *R *= 0.58, *P *= 0.06, respectively) (Figure 2A, B). No significant correlation was found between the responsiveness to trastuzumab and T-DM1 (*R *= 0.6, *P *= 0.07, Figure 2C).

### Trastuzumab- and T-DM1-mediated ADCC against HER2 positive breast cancer cells

Since it was previously shown that antibody-dependent cellular cytotoxicity (ADCC) has a key role in the *in vivo *effect of trastuzumab [10], we compared trastuzumab and T-DM1 in *in vitro *ADCC assay. Trastuzumab and T-DM1 evoked ADCC similarly on SKBR-3 and JIMT-1 breast cancer cells with dose-dependent cell death reaching approximately 70 to 85% killing using an effector/target ratio of 80:1. Therefore, conjugation with DM1 does seem not to alter the ability of trastuzumab to mediate ADCC (Figure 1C.).

### Effect of T-DM1 on JIMT-1 xenografts

**Figure 3:**
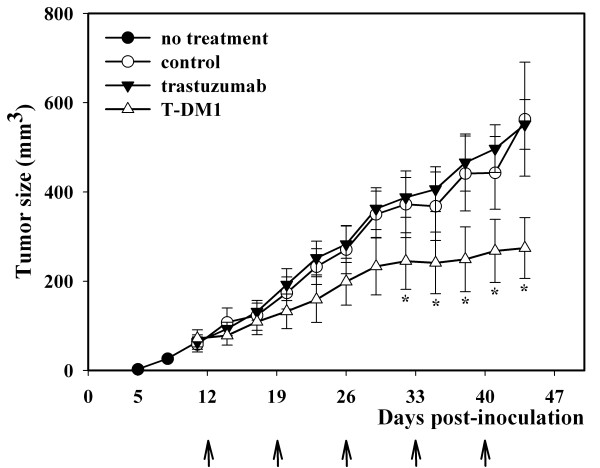
**Effect of T-DM1 on the growth of JIMT-1 xenografts**. SCID mice were injected s.c. with 5 × 10^6 ^JIMT-1 cells. Tumours were formed in all mice in 11 days (*n *= 18, mean tumour volume 64 ± 9 mm^3^). Thereafter, weekly treatments (arrows) with control antibody (rituximab, 5 mg/kg, i.p., white circle, *n *= 6), trastuzumab (5 mg/kg, i.p., black triangle, *n *= 6) or T-DM1 (15 mg/kg, i.v., white triangle, *n *= 6) were started on Day 12 and continued until the end of the experiment. A partial but significant tumour growth inhibition was seen by T-DM1 from days 32 to 44 (*, *P *< 0.05), while trastuzumab had no inhibitory effect on tumour growth.

The effects of T-DM1 were next studied *in vivo *using JIMT-1 xenograft models. Tumours were formed in all SCID mice inoculated with JIMT-1 cell suspension in 11 days (*n *= 18, mean tumour volume 64 ± 9 mm^3^). Thereafter, weekly treatments with trastuzumab and T-DM1 were started on Day 12 and continued until the end of the experiment. Rituximab was used as a negative control agent. While trastuzumab had no inhibitory effect on tumour growth, a partial but significant tumour growth inhibition was seen by T-DM1 from days 32 to 44 (*P *< 0.05) (Figure 3.).

### The effect of T-DM1 on the formation of JIMT-1 xenografts

**Figure 4:**
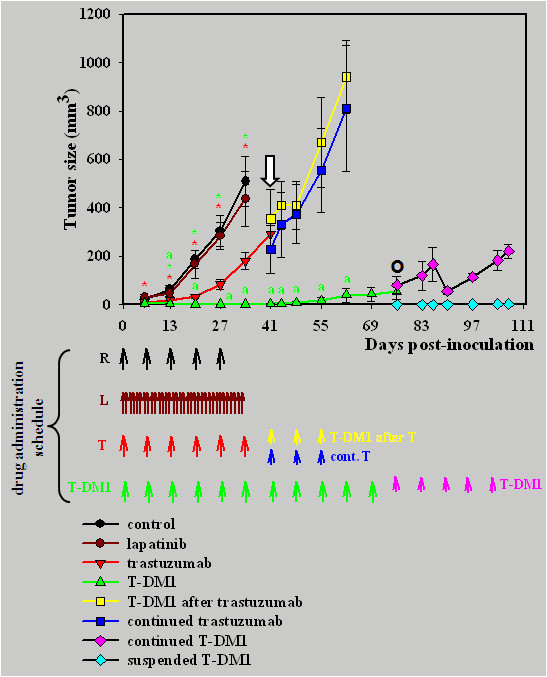
**Effect of T-DM1 on the formation of JIMT-1 xenografts**. SCID mice injected s.c. with 5 × 10^6 ^JIMT-1 cells were treated on a weekly basis with control antibody (rituximab, 5 mg/kg, i.p., black circle, *n *= 7), trastuzumab (5 mg/kg, i.p., red triangle, *n *= 6), T-DM1 (5 mg/kg, i.v., green triangle, *n *= 6) or on a daily basis with lapatinib (100 mg/kg, p.o., brown circle, *n *= 7) starting from the day of tumour inoculation (Day 0). Drug administration schedule is shown by arrows. Lapatinib (brown circle) had no effect on tumour growth. Trastuzumab (red triangle) reduced the formation of tumours between days 6 and 34 compare to both control antibody and lapatinib (red stars, *P *< 0.05). In three out of the six trastuzumab treated mice, trastuzumab administration was suspended and switched to T-DM1 (5 mg/kg, i.v., yellow square) from the Day 41 (marked by white arrow) whereas it was continued for another three weeks in the other three (5 mg/kg, i.p., blue square). In this setting, T-DM1 was unable to inhibit tumour growth. The tumours started to grow in four T-DM1 treated mice (green triangle) from Day 55 onward, but remained non-palpable in two (2/6). T-DM1 administration of these two mice was suspended on Day 76 (marked by Ο) and the mice were followed up for more four weeks (pale blue square, *n *= 2). In these two mice, a very small palpable tumour (approximately 4 mm^3^) was detected in one mouse on Day 107 and residual cancer cells were found histologically. No tumour was detected in the other mouse and the histological examination of the tumour inoculation site suggested a complete cure. T-DM1 treatment of the other four mice was continued until the end of the experiment (5 mg/kg, i.v., pink square, *n *= 4). Overall, T-DM1 significantly inhibited the tumour formation from Day 13 to Day 34 in comparison to both control antibody and lapatinib (green stars, *P *< 0.05), and it had a significantly growth inhibition between days 13 and 62 compared to trastuzumab (green *a*, *P *< 0.05). Note that both trastuzumab and T-DM1 were used in a dose of 5 mg/kg.

In the next experiment, T-DM1 treatment was started at the time of JIMT-1 cell suspension inoculation to see its effect on tumour formation. The tumours in T-DM1 treated mice remained very small until Day 55 (16 ± 12 mm^3^). Thereafter, the tumours started to grow in four out of six mice but remained non-palpable in two (2/6). Residual cancer cells were found histologically in one of these two mice, and a complete cure was suggested in the other. Overall, the effect of T-DM1 on tumour growth was much stronger when compared to trastuzumab or lapatinib (*P *< 0.05) (Figure 4.). In this setting we also tested lapatinib, which had no inhibitory effect on tumour growth in comparison to the control (Figure 4.).

### Effect of T-DM1 on JIMT-1 xenografts pretreated with trastuzumab

T-DM1 was tested also on mice treated first with trastuzumab since tumour cell inoculation (Day 0). As also shown before [11], trastuzumab slowed down the formation of tumours between days 6 and 34 (*P *< 0.05), probably via ADCC. For half of the mice trastuzumab was discontinued and switched to T-DM1 from Day 41 onwards. In this setting, T-DM1 was unable to inhibit tumour growth (Figure 4.).

### T-DM1 evokes mitotic catastrophe

**Figure 5:**
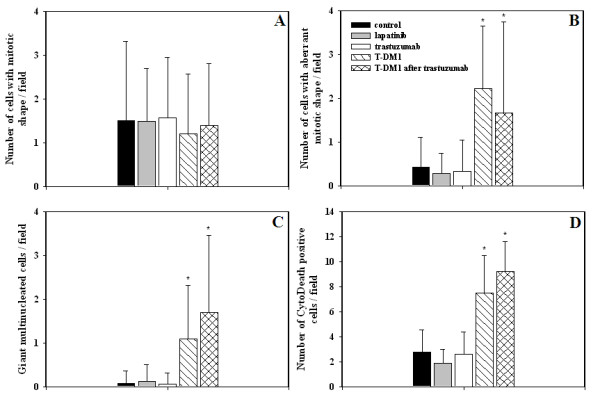
**T-DM1 induced mitotic catastrophe and apoptosis in JIMT-1 xenografts**. For histology, JIMT-1 tumours were obtained from SCID mice treated with control antibody (rituximab, black column), lapatinib (gray column), trastuzumab (white column), T-DM1 (hatched column) or T-DM1 after trastuzumab (cross-hatched column). Histological sections were stained with hematoxylin and eosin. When enumerating the number of mitoses with normal morphology, no differences were found between the samples (**A**). In contrast, a significantly higher number of cells with aberrant mitotic morphology was detected in T-DM1 treated tumours (**B**) (*, *P *< 0.05). The number of giant multinucleated cells was also increased in T-DM1 treated samples (**C**) (*, *P *< 0.05). Notably, higher number of cells with aberrant mitotic morphology and higher number of giant multinucleated cells were detected in the samples whose trastuzumab treatment was changed to T-DM1 (**B-C**) (*, *P *< 0.05). CytoDeath staining was used to detect the apoptotic cells in the histological sections. Significantly increased number of apoptotic cells was found in the T-DM1 treated samples and also in the in the samples whose trastuzumab treatment were changed to T-DM1 (**D**) (*, *P *< 0.05). Note that CytoDeath positive cells are plotted on different scales.

**Figure 6:**
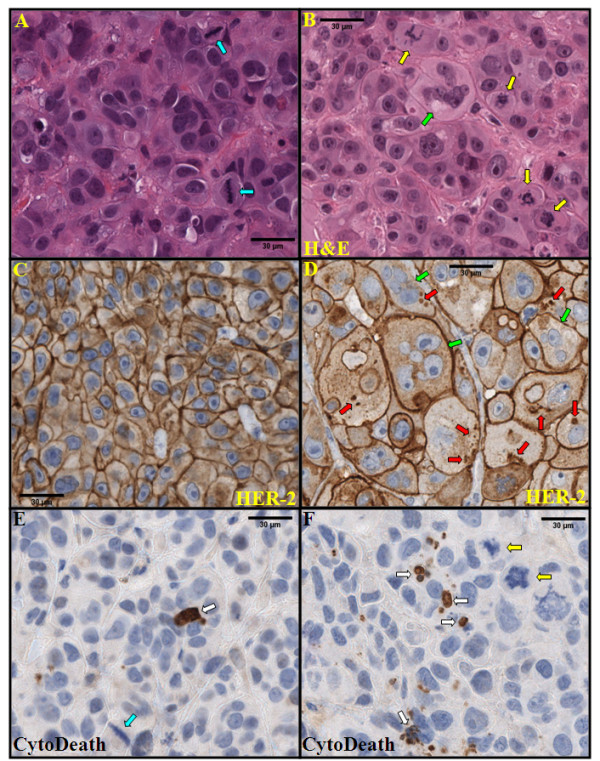
**Histological characterization of T-DM1 induced changes in JIMT-1 xenografts**. JIMT-1 xenografts were obtained from mice treated with trastuzumab (**A, C, E**) or T-DM1 (**B, D, F**). Histological sections were stained with hematoxylin and eosin (H&E, **A, B**). HER2 protein was visualized by HercepTest (**C-D**). Apoptotic cells were visualized by M30 CytoDeath antibody (**E-F**). High number of cells with aberrant mitotic shape (yellow arrow) and giant multinucleated cells (green arrow), which are the hallmarks of mitotic catastrophe, were seen in T-DM1 treated samples (**B, D, F**). Furthermore, higher number of apoptotic cells was found in the T-DM1 treated samples than in the trastuzumab treated ones (white arrow, **E **and **F**, respectively). Similar number of cells with normal mitotic shape (blue arrow) was found in trastuzumab and T-DM1 treated samples (**A**). Tumour cells retained their HER2 positivity after long-term trastuzumab (**C**) or T-DM1 treatment (**D**), approximately 9 and 15 wks, respectively. HER2 positive intracytoplasmic granules (red arrow) were seen in T-DM1 treated tumours (**D**), while not in trastuzumab treated ones (**C**). Giant multinucleated cells were strongly positive for cell membrane HER2 protein (green arrow, **D**).

Histological sections of the xenograft tumours were prepared and stained with hematoxylin and eosin. Enumeration of mitoses with normal morphology revealed no differences between trastuzumab and T-DM1 treated JIMT-1 xenografts (Figures 5A and 6A). In contrast, we detected a significantly higher number of cells with aberrant mitotic morphology in T-DM1-treated tumours (*P *< 0.05) (Figures 5B and 6B). In line with this observation, the number of giant multinucleated cells (GMCs) was increased in T-DM1 treated samples (*P *< 0.05) (Figures 5C and 6B, D). Aberrant mitosis and GMCs are hallmarks of mitotic catastrophe. We observed a higher number of cells with aberrant mitotic morphology and higher number of GMCs also in the tumour samples whose trastuzumab-treatment were changed to T-DM1 (*P *< 0.05) (Figure 5B, C.).

### T-DM1 evokes apoptosis

Apoptotic cells were detected using CytoDeath staining, which localizes a caspase-activated breakdown product of cytokeratin subtype 18. In this analysis we found significantly increased numbers of apoptotic cells in the T-DM1 treated samples in comparison to the trastuzumab treated ones (*P *< 0.05) (Figures 5D and 6E, F). We detected higher numbers of apoptotic cells also in the samples whose trastuzumab-treatment were changed to T-DM1 (*P *< 0.05) (Figure 5D.). It is also noteworthy that most of the cells with aberrant mitotic morphology were CytoDeath negative (Figure 6F).

### Expression of HER2 protein on trastuzumab and T-DM1 treated xenografts

Immunohistochemical staining localizing intracellular and extracellular epitopes of the HER2 protein was performed (using HercepTest and SP3 antibodies). No major qualitative changes in the cell membrane staining of HER2 expression were observed. Virtually all tumour cells showed strongly positive cell membrane staining reactions for both intracellular and extracellular epitopes (data not shown). However, it is noteworthy that staining positive intracytoplasmic granules were seen only in T-DM1 treated tumours. Cells undergoing mitotic catastrophe were strongly positive for cell membrane HER2 protein (Figure 6C, D).

## Discussion

Trastuzumab-DM1 (T-DM1) is a new antibody-drug conjugate (ADC) developed for the treatment of HER2 positive cancer. In the present study we showed that long-term T-DM1 treatment may have two mechanisms of action. In addition to induction of apoptotic cell death [25], histopathological and immunohistochemical examination of the JIMT-1 xenograft tumours revealed aberrant mitotic figures and giant multinucleated cells (GMC), which are hallmarks of mitotic catastrophe (MC) [33,34]. To the best of our knowledge, this is the first report which shows that T-DM1 evokes mitotic catastrophe on cancer cells *in vivo*. This observation is in line with the previous report that unconjugated maytansine caused multinucleation of leukemic cells *in vitro *[41].

Our experiments showed that T-DM1 has a strong growth inhibitory effect on trastuzumab sensitive and resistant HER2 positive breast cancer cell lines *in vitro*. T-DM1 inhibited also the growth of breast cancer cell lines which are cross-resistant to trastuzumab and lapatinib, suggesting that T-DM1 can circumvent the cross-resistance phenomenon. We focused our studies on the JIMT-1 breast cancer cell line, which is unique because of having several co-existing trastuzumab resistance mechanisms, including an activating mutation of the *PIK3CA *gene, low expression of PTEN, high expression of NRG1, and moderate expression of HER2 receptor (despite gene amplification). These features are present at variable levels in other breast cancer cell lines, whereas JIMT-1 is unique in displaying all these factors at the same time [40].

Based on our observations, apoptosis and MC occur in the same tumour. Data from experimental docetaxel therapy suggest that mitotic arrest and apoptosis can be seen at high drug concentrations, whereas aberrant mitosis and multinucleation are observed at low concentrations [42]. We hypothesize that T-DM1 might also have this dual mechanism of cytotoxicity. A high concentration of free DM1 in the cytoplasm might cause rapid apoptosis with no or few MC histologically detectable. When the treatment target (like the JIMT-1 cell line) is not fully sensitive to T-DM1, mitotic catastrophe develops as a more chronic and mild late effect. The level of HER2 expression, internalization rate of HER2 - T-DM1 complexes, rate of lysosomal degradation or recycling, and potential efflux of free DM1 by ABC-transporters [26] all might define the degree of effect. These factors which probably differ from tumour to tumour (or even from cell to cell) might determine whether the response to T-DM1 is apoptosis or MC. Further investigations are needed to confirm this hypothesis.

After inoculation of JIMT-1 cells into SCID mice, we applied three different treatment strategies with T-DM1. First, T-DM1 had a partial but significant inhibitory effect on firmly established subcutaneous tumour nodules. Second, T-DM1 had an almost complete inhibitory effect on xenograft tumour formation when the drug administration was started at the time of tumour cell suspension inoculation. This was carried out to mimic the adjuvant post-operative therapy situation of chemotherapy naïve patients. Despite a very significant therapeutic effect, tumours started to grow after 15 weeks in four out of six mice. Residual cancer cells were found in the fifth mouse microscopically; however, a complete cure was seen in the sixth mouse. In the third therapeutic situation, T-DM1 given after trastuzumab was unable to inhibit tumour growth. These tumours were larger at the time of T-DM1 administration than the tumours in the first two treatment strategies. However, even in these tumours, T-DM-1 caused histologically detectable mitotic catastrophe and apoptosis. These observations suggest that efficacy of T-DM1 is largely dependent on tumour burden of the JIMT-1 xenograft model used.

Our experiments measuring antibody-mediated cellular cytotoxicity (ADCC) clearly indicated that the cytotoxic mechanism of released DM1 is not the only one for T-DM1. We showed that trastuzumab and T-DM1 evoked similarly effective ADCC on HER2 positive target cells. Because ADCC has an important role in the efficacy of trastuzumab *in vivo *[10,11], this could be one explanation for better efficacy of T-DM1 in a low tumour burden *in vivo *situation.

Finally, we reported immunohistochemical findings of HER2 expression after prolonged trastuzumab and T-DM1 treatments. First, we found no obvious changes in HER2 expression level when T-DM1 treated tumours were compared to those from trastuzumab treated or control animals. Second, intense (3+) staining reaction detected by antibodies against intracellular and extracellular epitopes of HER2 suggests that the majority of HER2 protein in JIMT-1 xenografts is intact after T-DM1 and T treatments. Thus, in the JIMT-1 model system, appearance of the truncated p95-HER2 protein is probably minimal and does not explain emerging resistance. Third, in tumours treated with T-DM1 we found an accumulation of HER2 staining positive intracellular granules of variable size. Morphologically these granules resembled enlarged lysosomes. This could be an indication of a defect in the intracellular trafficking of HER2 protein induced by T-DM1. These issues warrant further studies both for their biologic and clinical relevance.

## Conclusions

We investigated the effect and mechanism of action of a recently developed antibody-drug conjugate (trastuzumab-DM1, T-DM1). We found that T-DM1 is highly effective even on breast cancer cell lines cross-resistant to trastuzumab and lapatinib *in vitro*. Using a trastuzumab-resistant xenograft tumour model, we showed that trastuzumab-DM1 can induce both apoptosis and mitotic catastrophe *in vivo*. The latter is a previously undescribed mechanism of action of T-DM1.

## Abbreviations

ADC: antibody-drug conjugate; ADCC: antibody-dependent cellular cytotoxicity; BSA: bovine serum albumin; DMEM: Dulbecco's Modified Eagle Medium; FCS: fetal calf serum; FISH: fluorescence in situ hybridization; GAHIG: goat anti-human immunoglobulin; GMC: giant multinucleated cell; H&E: hematoxylin and eosin; i.p.: intraperitoneally; i.v.: intravenously; LDH: lactate dehydrogenase; MC: mitotic catastrophe; SCID: severe combined immunodeficient; T-DM1: trastuzumab-derivative of maytansine 1.

## Competing interests

The authors declare that they have no competing interests.

## Authors' contributions

MB, MT and JI conceived and designed the experiments, MB and KK performed the experiments, MB and JI analyzed data, and MB and JI wrote the paper.
